# Optimization, characterization, and protective potentials of *Periploca angustifolia* extract against cadmium toxicity using response surface methodology as an optimizing tool

**DOI:** 10.3389/fphys.2026.1756889

**Published:** 2026-04-15

**Authors:** Imen Ben Abdelmalek, Khaled Athmouni, Saida S. Ncibi, Ghadah Khaled Yousuf, Maha Smaouia, Hanen Enneb, Abdelfattah El Feki

**Affiliations:** 1 Department of Biology, College of Science, Qassim University, Buraydah, Saudi Arabia; 2 Laboratory of Marine Biodiversity and Environment (LR18ES/30), University of Sfax, Sfax, Tunisia; 3 Laboratory of Animal Ecophysiology, Faculty of Sciences of Sfax, University of Sfax, Sfax, Tunisia; 4 Biology Department - Science College- Jazan University, Jazan, Saudi Arabia; 5 Biology Departement, College of Sciences, University of Hafr Al Batin, Hafr AlBatin, Saudi Arabia; 6 Dryland Farming and Oasis Cropping Laboratory, Arid Land Institue, University of Gabes, Medenine, Tunisia

**Keywords:** antioxidant activities, cadmium toxicity, characterization, microwave-assisted extraction, optimization, *P. angustifolia* roots

## Abstract

**Introduction:**

Phenolic compounds isolated from medicinal plants are used against several physiological disorders.

**Methods:**

In this investigation, microwave-assisted extraction parameters were optimized using response surface methodology (RSM) to determine the optimal extraction conditions for phenolic compounds from *Periploca angustifolia* extract (PAPE). The phenolic extract was characterized by nuclear magnetic resonance (NMR) spectroscopy. The antioxidant activity of the extract was evaluated using standard antioxidant assays. In addition, the protective effect of the phenolic extract was evaluated against cadmium toxicity.

**Results and discussion:**

Our analysis showed that the optimum extraction conditions were an irradiation time of 10 min, an extraction temperature of 100 °C, and a water-to-raw-material ratio of 30 mL/g. HPLC analysis indicated that PAPE contained quercetin, catechin, and caffeic acid. NMR (1D and 2D) spectra confirmed the compounds identified by HPLC. Antioxidant assays showed that PAPE exhibited a strong free-radical scavenging ability. *In vivo* analysis showed that PAPE produced a significant (*p* < 0.01) protective effect against oxidative damage induced by cadmium exposure in rat testicles. In conclusion, PAPE demonstrated strong antioxidant potential that is likely attributable to the active compounds present in the extract.

## Introduction

The toxic effects of cadmium (Cd) affect various tissues, including the kidney, liver, and lungs (Genchi et al., 2020). Cd exposure causes multiple injuries to the reproductive system (Kumar and Sharma, 2019). Numerous studies have established that the human testis exhibits heightened susceptibility to Cd, leading to toxic effects in the male reproductive organs, with pronounced impacts on testicular tissue and sperm quality. Natural phenolic compounds are used as therapeutics against various diseases that are associated with oxidative stress (Jomova et al., 2025). These natural molecules have diverse biological activities (Liu et al., 2024; Ayubi et al., 2025). Several studies indicate that phenolic compounds can inhibit acute or chronic human maladies (Tlili et al., 2018). In this context, several mechanisms have been proposed to explain the relationship between phenolic action and biological diseases. Phenolic compounds reduce the damage caused by oxidative stress, which is associated with their ability to scavenge reactive oxygen species (ROS) produced in organismal cells (Muscolo et al., 2024; Edo et al., 2025). These species cause numerous harmful biological effects, such as lipid peroxidation, DNA damage, and protein oxidation (Wang et al., 2025). In recent years, several studies have focused on the application of natural molecules against metal toxicity (Alcântara et al., 2019; Ceramella et al., 2024). In this regard, several authors have found that phenolic molecules, as natural compounds, exhibit potent antioxidant potential (Bouymajane et al., 2024; Zannou et al., 2024). Recently, studies have shown that phenolic compounds strongly inhibit ROS-induced oxidative damage (Altamim, 2025) and enhance the expression of antioxidant enzymes (Tofan et al., 2025). Catechin, quercetin, and kaempferol are major flavonoids in medicinal plants and show notable antioxidant and radical-scavenging activity. Therefore, developing a simple and efficient method to extract phenolic compounds from various medicinal plants is important (Alara et al., 2018).

*Periploca angustifolia* is an endemic medicinal plant frequently used in traditional medicine in Tunisia (Athmouni et al., 2017). In this context, Athmouni et al. (2017) found that *P. angustifolia* leaf extract contains several phenolic compounds. The *P. angustifolia* phenolic extract exhibited a strong ability to protect liver tissue against Cd exposure (Athmouni et al., 2017). Several studies have shown significant pastoral, ecological (anti-erosive), and medicinal (infusion hypotensive) values of *P. angustifolia* (Diwani et al., 2020). Limited information is available on the protective action of phenolic compounds isolated from *P. angustifolia* roots against Cd toxicity. This study is the first to analyze the protective effect of phenolic compounds isolated from *P. angustifolia* against Cd-induced testicular damage.

This study was designed to evaluate the effects of the irradiation time, temperature, and liquid–solid ratio on the phenolic compounds isolated from *P. angustifolia* roots. Moreover, in the present work, we aimed to assess the chemical characterization and the possible antioxidant and protective potential of the phenolic extract isolated from *P. angustifolia* against Cd-induced oxidative stress in rat testicles.

## Materials and methods

### Material and chemicals

The plant sample was collected from Djebel Bouramli (Gafsa, South Tunisia) in May 2014. This plant was identified by Professor Mohamed Chaieb, Laboratory of Biology, Sfax Faculty of Sciences. The herbarium voucher specimen was deposited in the Bioprocess Laboratory, Center of Biotechnology of Sfax, Tunisia. The roots were washed with tap water and then peeled. Then, the roots were cut and dried at the optimal temperature (25 °C) for 1 week. The dried material was powdered using a mill.

## Microwave-assisted extraction and quantification

### Extraction of the phenolic compound

The microwave-assisted extractions of phenolic compounds from *P. angustifolia* extract (PAPE) were performed using instruments from Shanghai SINEO Microwave Chemical Technology Co., Ltd. (China), equipped with a non-contact infrared temperature sensor. The temperature was controlled by variable microwave irradiations at 400 W, and the samples cooled by a nitrogen current at the end and stirred at regular intervals to ensure homogeneous exposure to microwave irradiation. After extraction, the mixture was cooled to room temperature and centrifuged at 3,250 g for 10 min. The phenolic extract was then filtered using microporous membranes (0.45 µm) and evaporated by rotary evaporation at 45 °C–55 °C. The total phenolics were quantified using the method described by Kim et al. (2003).

### Microwave-assisted extraction of the phenolic extract

The factorial design plan (2^3^) was used in this study to determine the influence of irradiation time, temperature, and liquid–solid ratio on the phenolic extraction procedure (Table 1). In this study, the coded variables were defined as follows: **X**_**1**_ = irradiation time, **X**_**2**_ = temperature, and **X**_**3**_ = liquid–solid ratio. The Box–Behnken design was used as an optimization plan in this study.

The different experimental runs are shown in Table 2. The phenolic content was considered the response variable. In this study, the phenolic content was expressed using the following Equation 1:

$$Y=-802.693+4.81345\times X_{1}+16.7581\times X_{2}+2.39815\times X_{3}-0.20921\times X_{1}^{2}+0.00455\times X_{1}\times X_{2}+0.0021\times X_{1}\times X_{3}-0.0870525\times X_{2}^{2}+0.00835\times X_{2}\times X_{3}-0.0548275\times X_{3}^{2}.$$ (1)

**TABLE 1:** Factor levels for a 2^3^ factorial design.

Factor	Parameter	Coded level		
		Low	Center	High
X_1_	Irradiation time	5	10	15
X_2_	Temperature	90	100	110
X_3_	Liquid–solid ratio	20	30	40

**TABLE 2:** Box–Behnken design matrix of the three variables in coded units and the response values for the extraction rate of the phenolic extract.

Run	Irradiation time (min)	Temperature (°C)	Liquid–solid ratio (mL g^-1^)	Total phenolic content (mg GAE/g extract)	
				Actual	Predicted
1	10	100	30	82.45	82.64
2	10	90	20	74.12	73.20
3	10	90	40	71.45	70.82
4	10	100	30	81.56	82.64
5	10	110	40	64.45	65.37
6	10	100	30	84.02	82.64
7	10	100	30	83.42	82.64
8	10	110	20	63.78	64.41
9	5	100	40	65.41	65.73
10	5	90	30	66.45	66.76
11	15	90	30	76.54	77.78
12	15	110	30	71.41	71.10
13	5	100	20	66.04	66.65
14	5	110	30	60.41	59.18
15	10	100	30	81.74	82.64
16	15	100	20	78.23	77.91
17	15	100	40	78.02	77.41

Here, **Y** is the total phenolic content (TPC) obtained by the regression model. **X**_**1**_, **X**_**2**_, and **X**_**3**_ are the coded variables.

### HPLC analysis

The phenolic compounds extracted from *P. angustifolia* roots were identified using high-performance liquid chromatography with diode array detection (HPLC–DAD) as the analysis method. The details of this method were published in Athmouni et al. (2017). The phenolic compounds of *P. angustifolia* were identified using an HPLC system (consisting of a vacuum degasser, an autosampler, and a binary pump with a maximum pressure of 400 bar; Agilent 1260 Infinity HPLC System, Agilent Technologies, Germany) equipped with a reversed phase C18 analytical column (4.6 x 100 mm and 3.5 µm particle size; Zorbax Eclipse XDB C18). In HPLC–DAD, the limit of detection (LOD) and limit of quantification (LOQ) define the method’s sensitivity, which are often measured by signal-to-noise (S/N) ratios of 3:1 for LOD and 10:1 for LOQ. The DAD detector was set to a scanning range of 200 nm–400 nm. Column temperature was maintained at 25 °C. The injected sample volume was 2 µL, and the flow-rate of thw mobile phase was 0.4 mL/min. Milli-Q water that consisted of 0.1% formic acid was taken as mobile phase B, and methanol was taken as mobile phase A. The optimized gradient elution was illustrated as follows: 0 min–5 min, 10%–20% A; 5 min–10 min, 20%–30% A; 10 min–15 min, 30%–50% A; 15 min–20 min, 50%–70% A; 20 min–25 min, 70%–90% A; 25 min–30 min, 90%–50% A; 30 min–35 min, return to initial conditions. Identification analysis was carried out by comparing their retention time with those obtained from the extract. For the quantitative analysis, a calibration curve was obtained by plotting the peak area against different concentrations for each identified compound at 280 nm: the obtained curves for all the identified compounds showed good linearity (with an average of *R* ^2^ = 0.99): Y = 22.456X + 5.63 for quercetin, Y = 23.642X + 3.2333 for caffeic acid, and Y = 3.6327X + 1.7743 for catechin.

### Nuclear magnetic resonance spectroscopy

The NMR (1D and 2D) spectra were recorded on a Bruker 400 MHz spectrometer operating at 400 and 100 MHz for ^1^H and ^13^C NMR, respectively. Chemical shifts (δ) are quoted in parts per million and are referenced to tetramethylsilane (TMS) as the internal standard.

### Antioxidant activity assay of the PAPE

Different tests of the antioxidant capacity were carried out to determine the ability of PAPE to scavenge free radicals such as the 2, 2 diphenylpicrylhydrazyl (DPPH•) and 2,2′-azinobis-(3 ethylbenzothiazoline-6 sulfonic acid) (ABTS•+) radicals.

In this study, the method described by Öztürk et al. (2011) was utilized to evaluate the capacity of PAPE to chelate DPPH^•^ radicals in the reaction solution. In brief, 4 mL of a methanol solution of DPPH (0.1 mM) was mixed with 1 mL of PAPE at different concentrations (0 mg mL^-1^–1 mg mL^-1^). Then, the reaction mixture was incubated in a dark room for 30 min. The results were measured at 517 nm. The following equation was applied to determine the percentage of DPPH• scavenging (Equation 2):

$$\%\text{ DPPH scavenging}=\left[\frac{\left(A_{\text{control}\;}-A_{\text{test}}\right)}{A_{\text{control}}}\right]\times 100.$$ (2)

Here, A_control_ is the absorbance of the control reaction, and A_test_ is the absorbance of the extract reaction.

The potential of PAPE to inhibit free radicals in the reaction mixture was also evaluated against ABTS^•+^ free radicals (Ozgen et al., 2006). The ABTS^•+^ free radical solution was prepared using 2.45 mM potassium persulfate as the oxidant and ABTS (7 mM in 20 mM sodium acetate buffer; pH 4.5). The reaction mixture was incubated in the dark for 12 h–16 h at room temperature. The absorbance of the reagent solution was calibrated at 0.7 ± 0.01. The absorbance was measured at 734. A volume of 20 μL of samples at different concentrations was mixed with 3 mL of ABTS reagent and incubated at 30 °C for 30 min. Radical scavenging activity against ABTS free radicals was measured according to the following Equation 3:

$$\%\text{ Inhibition}=\left[\frac{\left(A_{\text{control}}-A_{\text{test}}\right)}{A_{\text{control}}}\right]\times 100.$$ (3)

Here, A_control_ is the absorbance of the control reaction, and A_test_ is the absorbance of the extract reaction.

### *In vitro* lipid peroxidation inhibition assay

The ability of PAPE to inhibit lipid peroxidation was evaluated according to the method described by Halliwell and Gutteridge (2007). The rat liver homogenate was prepared by dissolving 10% of rat liver in 0.15 M potassium chloride. Then, a volume of 0.5 mL of the liver homogenate and 1 mL PAPE at different concentrations were mixed. Ferrous sulfate (50 μL, 0.07 M) was added to the solution mixture to stimulate lipid peroxidation in liver tissue. The reaction mixture was incubated at room temperature for 30 min. After incubation, thiobarbituric acid (TBA) (50 μL, 0.8% in 1.1% SDS) was added. Finally, the reaction mixture was incubated in boiling water for 15 min. The percentage of inhibition was calculated according to the following formula (Equation 4):

$$\%\text{ Inhibition}=\left[\frac{\left(A_{\text{control}}-A_{\text{test}}\right)}{A_{\text{control}}}\right]\times 100.$$ (4)

Here, A_control_ is the absorbance of the control reaction, and A_test_ is the absorbance of the extract reaction.

### *In vitro* protein glycation

In this study, the method described by Vinson and Howard (1996) was utilized to evaluate the capacity of PAPE to protect proteins against the glycation phenomenon. The protein solution was prepared to dissolve 7 mg mL^-1^ bovine serum albumin (BSA) in 50 mM phosphate buffer (pH 7.4) containing 0.02% (w/v) sodium azide. Then, the protein solution was mixed with PAPE at different concentrations (10, 20, 50, and 100 μg mL^-1^) and incubated at room temperature for 30 min. Finally, glucose (25 mM) and fructose (25 mM) solutions were added to the reaction mixture. The results were determined using a fluorescence reader with an excitation wavelength of 350 nm and an emission wavelength of 450 nm. Quercetin was used as the standard (20 μg mL^-1^). The results were expressed as arbitrary units of fluorescence from the glycated protein.

### *In vivo* protective effect of PAPE

The experimental protocol was approved by the Local Ethics Committee for Animal Experiments in Tunisia and was performed according to the ethical principles, institutional guidelines, and the International Guide for the Use of Animals in Biomedical Research.

In this study, Wistar rats (male) purchased from the Tunisian Central Pharmacy (180 ± 20 g) were used to evaluate the protective activity of PAPE against Cd exposure. The animals were fed a commercial pellet diet (Socco, Sfax, Tunisia) and water *ad libitum* and maintained at a temperature of 25 °C ± 3 °C with a normal 12 h light/dark cycle. After acclimatization, the animals were randomly divided into four groups (n = 5): group I, control; group II: rats receiving PAPE (200 mg/kg/day); group III: rats receiving Cd chloride (Cd; 0.7 mg/kg/day), and group IV: rats treated by PAPE followed by CdCl_2_ (200 and 0.7 mg/kg/day, respectively).

After 1 month of treatment, the animals were deprived of food overnight, anesthetized by exposure to diethyl ether, and then sacrificed by cervical decapitation. The testicle tissue was dissected, washed in ice-cold saline, patted dry, and weighed. A small portion of the tissue was stored in 10% formalin for histopathological examination. From the remaining tissue, 1 g was weighed and homogenized in 2 mL PBS buffer (pH 7.4) and centrifuged for 15 min at 9,000 rpm at 4 °C. The supernatants were used to perform some biochemical tests.

### Testicular antioxidant status

The ability of the testicle supernatant from each group to inhibit the photoreduction of nitroblue tetrazolium (NBT) into blue formazan was used to determine superoxide dismutase (SOD) activity. The assay was performed in 50 mM PBS (phosphate-buffered saline) (pH 7.4) containing 13 mM methionine, 0.1 mM EDTA, 2 µM riboflavin, and 75 µM NBT. Activity was expressed as units/mg of protein, with one unit defined as the amount inhibiting the photoreduction of NBT by 50%. The test was performed at 25 °C.

The method described by Aebi (1984) was used to evaluate the catalase (CAT) activity of each group by measuring the decrease in H_2_O_2_ absorbance at 240 nm for 1 min. The results were expressed as µM of H_2_O_2_/min/mg of protein.

The method described by Flohé and Günzler (1984) was used to determine the glutathione-peroxidase (GPx) activity. In brief, a volume of 250 µL of samples from each group was mixed with GSH (0.35 mM). The reaction started with the addition of H_2_O_2_ (0.2 mM). The result was measured at 412 nm after terminating the reaction with Ellman’s reagent. The activity was expressed as µM of GSH oxidized/min/mg protein.

### Testicular oxidative stress markers

The lipid peroxidation content of each sample was determined using the method described by Esterbauer (1993). In brief, 125 µL of the homogenate testicle of each group was mixed with 125 µL of trichloroacetic acid (20%) containing butyl hydroxytoluene (1%). After centrifugation (1,000 g, 10 min at 4 °C), 200 µL of the supernatant was mixed with 40 µL of HCl (0.6 N) and 160 µL of thiobarbituric acid (120 mM) in 25 mM Tris buffer (pH = 7.4). The mixture was heated at 80 °C for 10 min, and the optical density of the solution was measured at 530 nm. The TBARS concentration was calculated using an extinction coefficient of 1.56 × 10^5^ mM^-1^ cm^-1^.

The spectrophotometric method was used to evaluate the protein carbonyl concentration in the testicle homogenate (Levine et al., 1990). The result was expressed as nM mg^-1^ protein.

### Testicular histopathological examination

Parts of testicle tissue obtained from each animal were fixed in 10% formalin solution, dehydrated in ascending grades of alcohol, and embedded in paraffin. Sections at four-micron thickness were taken, stained with hematoxylin and eosin (H&E), and examined under a light microscope to detect degeneration, vacuolization, and necrosis in the studied organ.

### Statistical analysis

Statistical analysis was performed on SPSS software (version 20). Data were expressed as the mean ± standard deviation and analyzed by one-way analysis of variance (ANOVA). The significance level was determined (*p* < 0.05), and significant differences were separated using Duncan’s multiple range test (DMRT).

## Results and discussion

### Optimization of microwave-assisted extraction of PAPE

**FIGURE 1:**
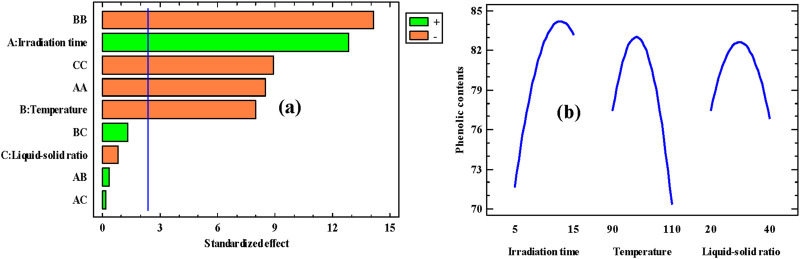
**(a)** Standardized Pareto chart for extraction efficiency and **(b)** effects of variations in the three parameters on phenolic extraction.

The colorimetric method used to estimate the phenolic content in PAPE has several important limitations, First, it lacks specificity because the Folin–Ciocalteu reagent reacts with all reducing substances, not only phenolic compounds. Therefore, while this method is simple, rapid, and inexpensive, it should ideally be complemented with more specific analytical methods. Table 2 shows the TPC of PAPE. Our results showed that PAPE is rich in phenolic components. Based on the analysis, the amount of PAPE varied from 61.12 to 83.45 mg GAE/g dw. Jaouhari et al. (2025) indicated that microwave-assisted extraction is the most effective method used for isolating these phenolic compounds from medicinal plants. In this context, Xi and Yan (2017) and Lozano Pérez et al. (2024) found that the microwave-assisted extraction method may be influenced by several factors, including irradiation time, temperature, and liquid–solid ratio. In this study, the *P. angustifolia* phenolic extract was optimized before *in vitro* antioxidant exploration. The parameter effects and their interactions in decreasing order of importance are given in Figure 1. In this study, there are three main effects (A: irradiation time, B: temperature, and C: liquid–solid ratio). Figure 1a shows that the effect of temperature (parameter B) is very important. In addition, irradiation time is an essential parameter affecting the extraction of phenolic compounds. Based on the analysis, the phenolic content of *P. angustifolia* extract significantly increased with extraction temperatures. The amount of phenolic extract was significantly enhanced with increasing temperature from 90 °C to 100 °C. Further increments in temperature beyond 100 °C induced a significant decrease. Alara et al. (2018) found that the yield of *Vernonia amygdalina* phenolic extract increased with higher extraction temperatures. It has additionally been found that the isolation of phenolic compounds from tea powders significantly increased with the elevation of the extraction temperature (Ismaiah et al., 2025). The same results were found by Kattour et al. (2025). Our results can be explained by the increased mass transfer of intracellular bioactive compounds at high temperatures. Recent studies have shown that elevated extraction temperature increase the solubility of the biological material and decrease the density and viscosity of the solvent (Xu et al., 2017).

Figure 1b presents the effect of irradiation time on the TPC. Based on the analysis, an increase in the TPC was observed from 5 to 10 min, and any further extension resulted in a reduction of the TPC of this extract. Moreover, the effects of temperature on the TPC of *P. angustifolia* roots are shown in Figure 1b. Microwave-assisted extraction enhances the recovery of phenolic compounds through rapid and uniform volumetric heating generated by dipole rotation and ionic conduction. This internal heating leads to localized pressure build-up within plant tissues, promoting cell-wall disruption and facilitating the release of intracellular phenolic constituents. Moreover, the elevated temperature improves solvent penetration, increases solubility, and accelerates mass transfer, thereby enhancing the extraction efficiency.

Moreover, the water- to-raw-material ratio was a crucial factor in the extraction of phenolic compounds. Our findings indicated that the phenolic content of *P. angustifolia* extract significantly increased with an increasing liquid–solid ratio from 20 to 30 mL g^-1^, but further increases beyond 30 mL g^-1^ did not enhance the yield. Our results can be explained by the inadequate stirring of a larger solvent volume during microwave application. In addition, Spigno et al. (2007) found that a larger solvent volume requires more absorption of microwave energy, which leads to insufficient leaching of the phenolic components. Similar results were found in *Sophora japonica* L (Xi and Yan, 2017). It has been reported that a high liquid–solid ratio improves the interaction between the sample and solvent matrix, thereby increasing the phenolic yield (Azahar et al., 2017). Increasing the solvent volume relative to the solid material enhances the concentration gradient between the plant matrix and the solvent, thereby improving mass transfer and the diffusion of phenolic components into the extraction medium. A higher solvent ratio also facilitates better penetration of the solvent into plant tissues and increases the solubility of the phenolic constituents.

**FIGURE 2:**
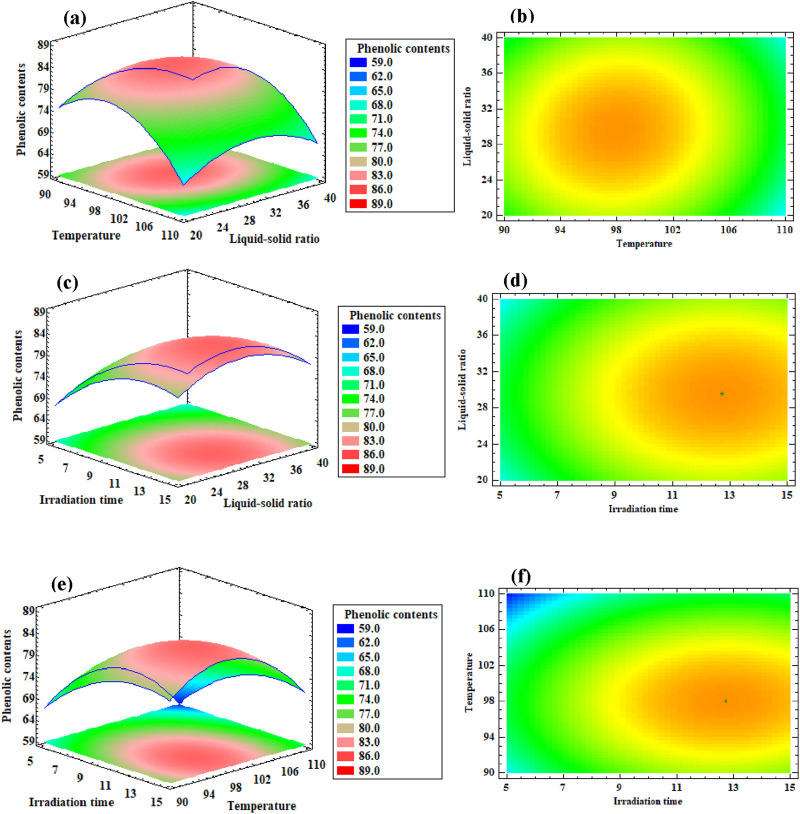
**(a)** The response surface plots of the effect of temperature, liquid solid ratio and their reciprocal interaction on phenolic content. Irradiation time was constant at 10 min. **(b)** 2D-contour line of the effect of temperature, liquid solid ratio and their reciprocal interaction on phenolic content. Irradiation time was constant at 10 min. **(c)** The response surface plots of liquid-solid ratio, irradiation time and their reciprocal interaction on phenolic content. Extraction temperature was maintained at 100 °C. **(d)** 2D contour line of liquid-solid ratio, irradiation time and their reciprocal interaction on phenolic content. Extraction temperature was maintained at 100 °C. **(e)** The response surface plots of temperature, irradiation time and their reciprocal interaction on phenolic content. Liquid solid ratio was fixed at 30 ml g^-1^. **(f)** 2D contour line of temperature, irradiation time and their reciprocal interaction on phenolic content. Liquid solid ratio was fixed at 30 ml g^-1^.

In addition, extraction efficiency was also affected by extraction time, which is one of the most important factors influencing the procedure. The content of PAPE increased significantly with increasing extraction time (Figure 1b). The effects of the liquid–solid ratio, extraction time, and temperature level on phenolic extraction are shown in Figure 2.

Table 3 shows the ANOVA for the experimental results of the optimization test. The *p*-value was 0.0011 for PAPE, indicating that the model was highly significant and suitable. However, there was no significant lack of fit (*p* > 0.05) for this model, indicating that it can be used to predict the responses. The coefficient of determination (R2) of the model was 0.9523, indicating that it sufficiently represented the real relationship between the independent and response variables.

**TABLE 3:** ANOVA for the effect liquid to material ratio, temperature and irradiation time on the content of PAPE using the quadratic response surface model.

Source	Df	PAPE content		
		Sum of squares	F-ratio	*p*-value
Model	9	70.562		0.0011
X_1_	1	68.2541	6.88	0.042
X_2_	1	4.6352	0.52	0.003
X_3_	1	23.745	2.35	0.002
X_1_^2^	1	118.963	12.56	0.012
X_1_X_2_	1	11.42	1.024	0.523
X_1_X_3_	1	1.5632	0.15	0.065
X_2_^2^	1	33.452	3.45	0.142
X_2_X_3_	1	0.0854	0.04	0.74
X_3_^2^	1	7.4526	0.72	0.356
Lack of lif	3	22.32	0.48	0.132

*p* < 0.01, highly significant; 0.01 ≤ *p* ≤ 0.05, significant; *p* ≥ 0.05, not significant.

### Preliminary characterization of PAPE

**FIGURE 3:**
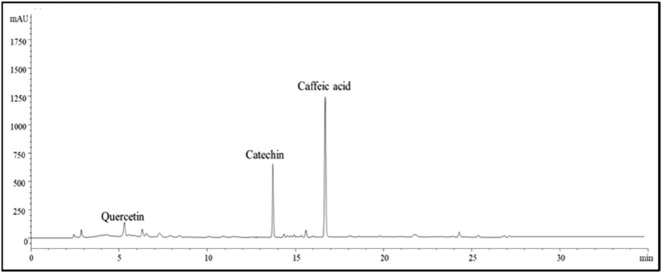
HPLC analysis at 280 nm of the *Periploca angustifolia* phenolic extract.

**FIGURE 4:**
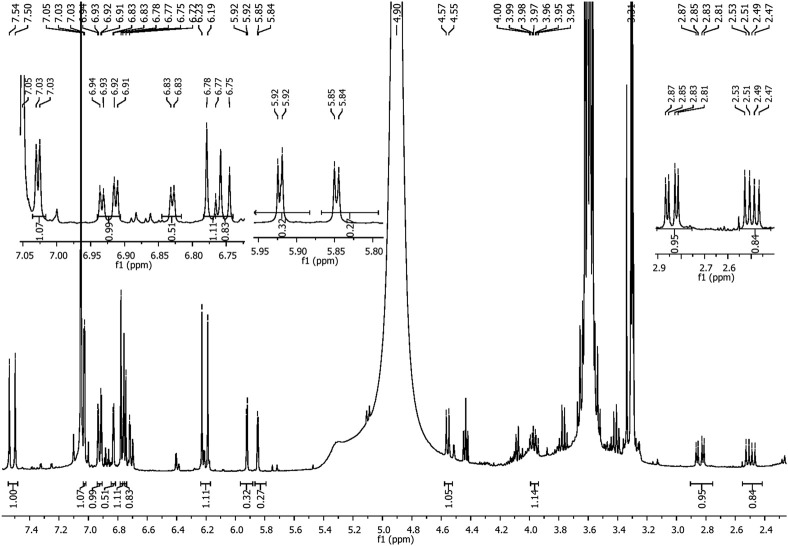
^1^H NMR spectra of caffeic acid and catechin (400 MHz, CD_3_OD).

**FIGURE 5:**
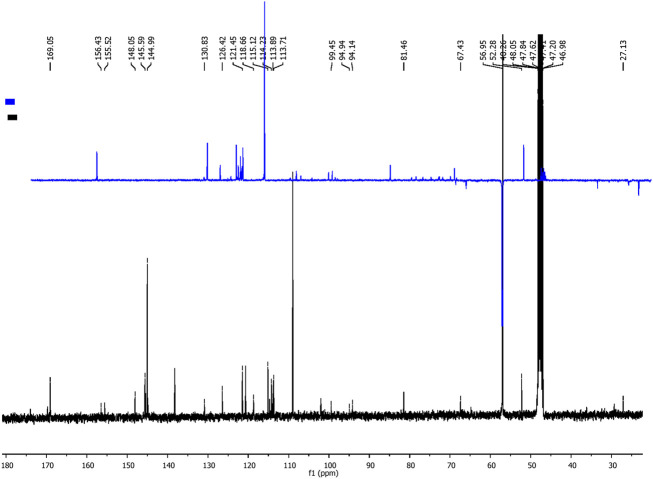
^13^C-NMR (broad-band and DEPT 135) spectra of PAPE fraction (400 MHz, CD_3_OD).

**FIGURE 6:**
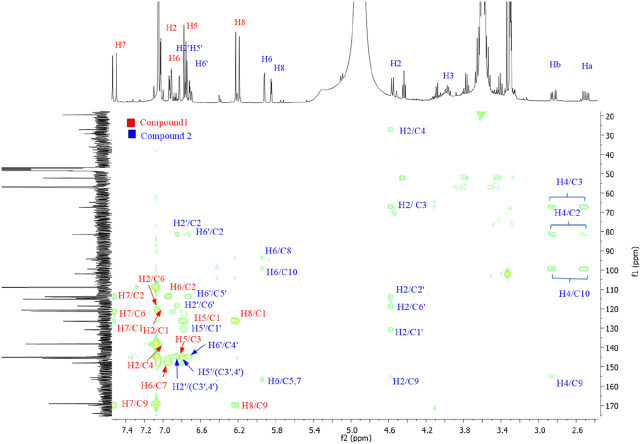
Representative HMBC spectra of PAPE fraction (400 MHz, CD_3_OD).

The Folin–Ciocalteu method typically has an LOQ for total phenolic content of approximately 0.5 μg/mL–10 μg/mL. In this study, a more sensitive technique, HPLC, was used. The phenolic composition of *P. angustifolia* roots was characterized by HPLC–DAD analysis. The analysis showed that the PAPE contained several flavonoid molecules, including catechin, caffeic acid, and quercetin (Figure 3). Catechin was the predominant compound in PAPE. Similarly, Diwani et al. (2020) reported a high content of catechin in *P. angustifolia* roots, and Athmouni et al. (2017) found a significant amount of catechin in phenolic extracts from *P. angustifolia* leaves. The NMR (1D and 2D) spectra were recorded on a Bruker 400 MHz spectrometer, operating at 400 and 100 MHz for ^1^H and ^13^C NMR. Chemical shifts (δ) are quoted in parts per million and are referenced to TMS as the internal standard. The NMR spectra (^1^H and ^13^C, respectively, Figures 4, 5) of our fraction showed the intense signals relative to caffeic acid and less intense signals related to catechin as the predominant compounds, along with weak signals due to other minor compounds, most likely quercetin. On the one hand, the ^14^H NMR spectrum of our fraction dissolved in CD_3_OD showed the presence of two intense doublets at 6.21 ppm (d, *J* = 15.9 Hz, 1H) and 7.52 ppm (d, *J* = 15.9 Hz, 1H) corresponding to H8 and H7, respectively, protons that are shown to be attached to the carboxyl group by HMBC (Figure 6), along with the presence of the three signals that correspond to aromatic protons (H2, H5, and H6). These signals confirm the presence of caffeic acid in our fraction (compound 1). On the other hand, the presence of two doublets of doublet at 2.50 and 2.84 ppm corresponds to the methylene protons Ha and Hb, respectively, and a multiplet centered at 3.94 ppm–4.00 ppm is attributed to the methine nuclei H3, which is J-coupled to the adjacent methylene protons H4 and to the methine H2 at 4.56 ppm, establishing long-range carbon/proton correlations ^2^ *J*_C–H_ and ^3^ *J*_C–H_ HMBC) (Figure 6; Table 4). In the high-field region, two doublets at 5.85 ppm (d, *J* = 2.3 Hz, 1H) and 5.92 ppm (d, *J* = 2.3 Hz, 1H) correspond to the meta-protons of A-ring H8 and H6, respectively, and the three-spin system at 6.71 ppm (dd, *J* = 8.2 and *J* = 1.9 Hz, 1H, H6′), 6.76 ppm (d, *J* = 8.2 Hz, 1H, H5′), and 6.83 ppm (d, *J* = 1.9 Hz, 1H, H2′) are consistent with the catechol protons of the B-ring. These signals showed the presence of catechin (compound 2) in our fraction.

**TABLE 4:** Correlations of carbon/proton ^2^J_C–H_ and ^3^J_C–H_ of compounds 1 and 2 (400 MHz, CD_3_OD).

Signals	Compound 1		Compound 2	
	Carbons δ (ppm)	Correlations	Carbons δ (ppm)	Correlations
1	C1 (126.4)	H2 (7.03), H5 (6.77), H6 (6.92), H7 (7.52), and H8 (6.21)	—	
2	C2 (113.7)	H2 (7.03) and H6 (6.92)	C2 (81.4)	H2’ (6.83), H3 (3.94–4.00), Ha (2.50), Hb (2.84), and H6’ (6.71)
3	C3 (144.9)	H2 (7.03) and H5 (6.77)	C3 (67.4)	H2 (4.56), Ha (2.50), and Hb (2.84)
4	C4 (148.0)	H2 (7.03), H5 (6.77), and H6 (6.92)	C4 (27.1)	H2 (4.56) and H3 (3.94–4.00)
5	C5 (115.1)	H6 (6.92)	C5 (156.4)	H6 (5.92), Ha (2.50), and Hb (2.84)
6	C6 (121.4)	H2 (7.03), H5 (6.77), and H7 (7.52)	C_6_ (94.9)	H8 (5.85)
7	C7 (145.5)	H6 (6.92) and H8 (6.21)	C7 (156.4)	H6 (5.92) and H8 (5.85)
8	C8 (114.2)	H7 (7.52)	C8 (94.1)	H6 (5.92)
9	C9 (169.0)	H7 (7.52) and H8 (6.21)	C9 (155.5)	H2 (4.56), Ha (2.50), and Hb (2.84)
10	—		C10 (99.4)	H3 (3.94–4.00), Ha (2.50), Hb (2.84), H6 (5.92), and H8 (5.85)
1′	—		C_1’_ (130.8)	H2 (4.56), H3 (3.94–4.00), H2’ (6.83), H5’ (6.76), and H6’ (6.71)
2′	—		C2’ (113.7)	H2 (4.56) and H6’ (6.71)
3′	—		C3’ (144.9)	H2’ (6.83) and H5’ (6.76)
4′	—		C4’ (144.9)	H2’ (6.83), H5’ (6.76), and H6’ (6.71)
5′	—		C5’ (114.7)	H6’ (6.71)
6′	—		C6’ (118.6)	H2 (4.56), H2’ (6.83), and H5’ (6.76)

### Antioxidant capacity of PAPE

**FIGURE 7:**
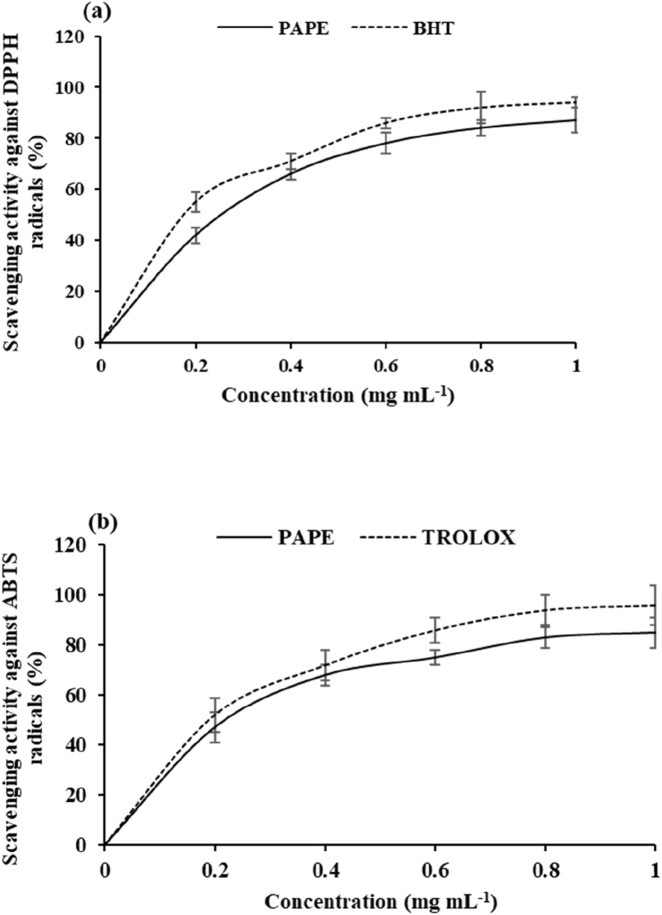
Scavenging effects of the *Periploca angustifolia* phenolic extract on **(a)** DPPH assay and **(b)** ABTS radical assay at different concentrations, compared with the respective standards (BHT and TROLOX). Values are presented as the mean ± SD (n = 3).

The antioxidant capacity of PAPE against DPPH and ABTS free radicals is shown in Figure 7. The antioxidant capacity values for the studied extract varied between 45.23% and 86.15% (Figure 7a). Athmouni et al. (2017) found that *P. angustifolia* methanolic extract showed a strong ability to scavenge the DPPH free radical. In addition, Diwani et al. (2020) found that condensed tannin isolated from *P. angustifolia* roots exhibited high capacity to inhibit DPPH radicals in the reaction mixture. Figure 7b shows the ability of PAPE to scavenge the ABTS•+ free radical, with scavenging activity values for the analyzed extract ranging from 37.21% to 82.45%. This activity can be attributed to the capacity of PAPE to donate hydrogen and scavenge free radicals. In addition, the antioxidant potential of phenolic compounds usually depends on the number of hydroxyl groups (Wang et al., 2017). These results are consistent with Chen et al. (2012), who reported that the highest antioxidant potential of phenolic compounds is associated with the lower bond dissociation energies (BDEs) of their OH groups. Phenolic compounds possess hydroxyl groups that are capable of donating hydrogen atoms or electrons to neutralize free radicals, thereby preventing oxidative damage. Our results showed that the PAPE fraction had a strong ability to scavenge DPPH and ABTS radicals. The ability of phenolic compounds to scavenge free radicals enables interaction with ROS, which can induce oxidative stress damage in tissues. Their redox properties allow them to act as reducing agents, metal chelators, and quenchers of singlet oxygen. Consequently, a higher total phenolic content is often correlated with stronger antioxidant capacity. This positive correlation indicates that phenolic constituents are major contributors to the overall antioxidant potential of this extract.

### Preventive action of PAPE against ferrous sulfate-enhanced lipid peroxidation *in vitro*

**FIGURE 8:**
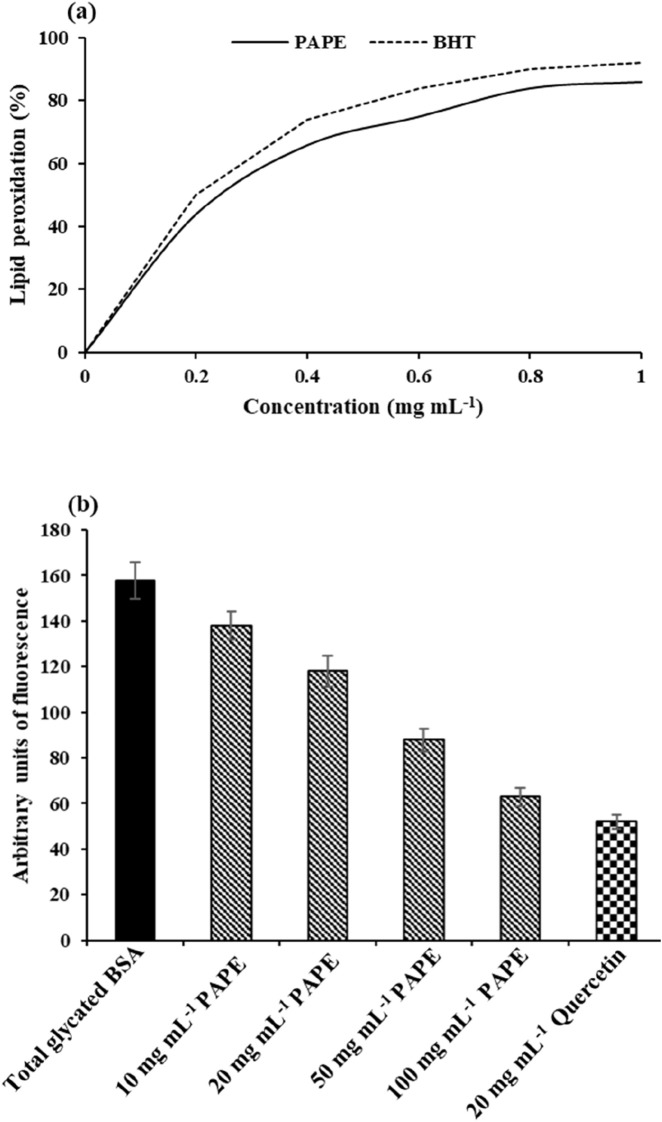
Effect of different PAPE concentrations on **(a)** lipid peroxidation and **(b)** protein glycation. BHT and quercetin were used as standards.

*P. angustifolia* phenolic extract inhibits ferrous sulfate-enhanced oxidative injuries in liver homogenates. Figure 8a shows that the inhibition percentage of lipid peroxidation values for the analyzed extract varied between 34.12% and 87.21%. In this context, Athmouni et al. (2017) found that the *P. angustifolia* methanolic extract inhibited Cd-induced lipid peroxidation in HepG2 cells. Similar to the present study, Rajesh et al. (2013) found that *Mesua ferrea* phenolic extract has a strong protective effect against ferrous sulfate-induced oxidative damage in liver homogenates. Phenolic extract protects lipids from oxidative damage primarily through their antioxidant mechanisms. They act as free radical scavengers by donating hydrogen atoms or electrons to lipid radicals, thereby terminating the chain reactions of lipid peroxidation. Additionally, phenolic compounds can chelate pro-oxidant metal ions such as Fe^2+^, which catalyze the formation of ROS that initiate lipid peroxidation. The phenolic extract effectively inhibits lipid peroxidation through these combined actions of (1) radical scavenging and (2) metal chelating.

### Preventive action of PAPE against protein glycation *in vitro*

The potential of PAPE to inhibit protein glycation was evaluated using a fluorescence method. Quercetin was used as the standard. A glycation-inducing reaction system containing bovine serum albumin, fructose, and glucose was used as the model. Figure 8b shows the percentage of inhibition of protein glycation. PAPE demonstrated strong inhibition of protein glycation at 10, 20, 50, and 100 μg·mL^−1^. In addition, this fraction showed a high capacity to inhibit protein glycation in the *in vitro* system. These results are consistent with observations by Da Silva Morrone et al. (2013), who reported that *Passiflora manicata* phenolic compounds have strong anti-glycation properties. Accordingly, the PAPE fraction can be further explored *in vitro* and in animal models.

### Protective effect of PAPE against CdCl_2_-induced oxidative stress in testicular tissue

Table 5 shows the protective effect of PAPE on Ca-induced oxidative damage in testicular tissue. Our analysis showed that Cd induced significant (*p* < 0.01) increases in MDA and PCO levels. Peana et al. (2023) found that Cd exposure enhanced MDA levels. In addition, Cd exposure significantly (*p* < 0.01) inhibited the antioxidant enzyme activities of CAT, SOD, and GPx in testicular tissue. The same results were reported by Hassanein et al. (2025), who indicated that Cd-induced oxidative stress was associated with decreases in GPx, CAT, Mn–SOD, and cytosolic CuZn–SOD. The protective action of PAPE was evaluated by treating rats with 100 mg kg-1 of the extract followed by Cd exposure (0.7 mg kg^-1^). Treatment with 100 mg kg^-1^ of PAPE significantly (*p* < 0.01) increased antioxidant enzymes and decreased MDA and PCO levels in testicular tissue. PAPE protects lipids against Cd-induced oxidative damage through multiple mechanisms. It scavenges ROS generated by Cd, donates electrons or hydrogen to neutralize free radicals, and chelates Cd ions to reduce their pro-oxidant activity. Together, these actions inhibit lipid peroxidation and stabilize cell membranes under Cd-induced oxidative damage. Moreover, PAPE protects enzymatic antioxidant systems, such as SOD, CAT, and GPx, by reducing oxidative damage and preventing enzyme inactivation. It scavenges ROS, thereby lowering the burden of free radicals that can damage these enzymes. Phenolic compounds can upregulate the expression or activity of antioxidant enzymes by modulating cellular signaling pathways, thereby enhancing the endogenous defense system.

**TABLE 5:** Effect of PAPE extraction against Cd-induced damage in the antioxidant and pro-oxidant status in rat testicular samples.

Parameters	Group 1	Group 2	Group 3	Group 4
SOD^α^	44.54 ± 3.65^d^	45.23 ± 2.54^a^	24.32 ± 2.63^b^	37.56 ± 1.85^c^
CAT^β^	35.63 ± 3.22^d^	34.21 ± 2.32^a^	19.74 ± 1.52^b^	30.12 ± 1.24^c^
GPx^γ^	321.23 ± 7.52^d^	320.75 ± 5.63^a^	288.31 ± 6.32^b^	214.63 ± 4.52^c^
MDA^λ^	0.56 ± 0.07^a^	0.53 ± 0.13^d^	2.56 ± 0.27^c^	1.43 ± 0.31^b^
Protein carbonyl^φ^	1.21 ± 0.25^a^	1.18 ± 0.45^d^	4.45 ± 0.22^c^	1.52 ± 0.14^b^

Means with different letters (a, b, c et d) indicate significant differences (p < 0.05) in ascending order.

α, U mg^-1^ protein.

β, µmoles/H_2_O_2_ consumed min^-1^ mg^-1^ of protein.

γ, nmoles GSH min^-1^ mg^-1^ of protein.

λ, nmoles of MDA g^-1^ of tissue.

φ, nmol mg^-1^ of protein.

### Effects of CdCl_2_ exposure on the testicular structure

**FIGURE 9:**
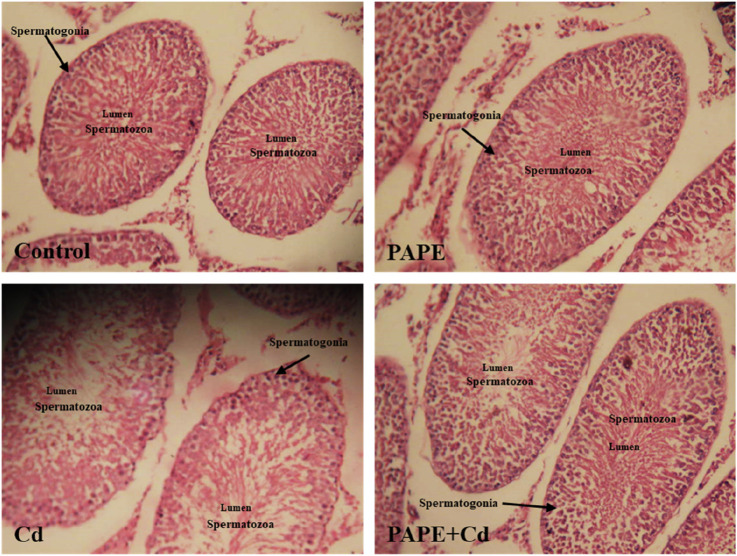
Histopathological examination of testicular sections of different groups of rats. Control groups; PAPE group, rats treated only with phenolic extract (100 mg/kg); Cd group, rats treated only with cadmium (0,7 mg/kg); PAPE + Cd group, rats treated with both phenolic extract and cadmium.

Figure 9 shows that Cd-exposure caused several alterations in testicular tissues, including necrosis and destruction of the testicular lumen, which inhibited spermatogenesis. The administration of PAPE preserved the morphology and also restored the spermatogenesis process. Hassanein et al. (2025) found that Cd exposure inhibited spermatogenesis in mice.

## Conclusion

In conclusion, this study demonstrates that optimizing the extraction parameters enhances the recovery of phenolic compounds, leading to improved antioxidant activity. Our analysis showed that the optimum extraction conditions were an irradiation time of 10 min, an extraction temperature 100 °C, and a water-to-raw-material ratio of 30 mL/g. Furthermore, the phenolic extract exhibited a protective effect against Cd-induced oxidative stress in testicular tissue, as evidenced by the preservation of lipid integrity and the maintenance of enzymatic antioxidant systems (SOD, CAT, and GPx). These findings indicate that phenolic compounds isolated from *P. angustifolia* roots have significant potential as natural antioxidants and protective agents against heavy metal-induced reproductive toxicity.

## Data Availability

The raw data supporting the conclusions of this article will be made available by the authors, without undue reservation.
